# Artificial Neuron using Vertical MoS_2_/Graphene Threshold Switching Memristors

**DOI:** 10.1038/s41598-018-35828-z

**Published:** 2019-01-10

**Authors:** Hirokjyoti Kalita, Adithi Krishnaprasad, Nitin Choudhary, Sonali Das, Durjoy Dev, Yi Ding, Laurene Tetard, Hee-Suk Chung, Yeonwoong Jung, Tania Roy

**Affiliations:** 1 NanoScience Technology Center, University of Central Florida, Orlando, Florida 32826 USA; 2 Department of Electrical and Computer Engineering, University of Central Florida, Orlando, Florida 32816 USA; 3 Department of Materials Science and Engineering, University of Central Florida, Orlando, Florida 32816 USA; 4 Department of Physics, University of Central Florida, Orlando, Florida 32816 USA; 5 Analytical Research Division, Korea Basic Science Institute, Jeonju, jeollabuk-do, 54907 South Korea

## Abstract

With the ever-increasing demand for low power electronics, neuromorphic computing has garnered huge interest in recent times. Implementing neuromorphic computing in hardware will be a severe boost for applications involving complex processes such as image processing and pattern recognition. Artificial neurons form a critical part in neuromorphic circuits, and have been realized with complex complementary metal–oxide–semiconductor (CMOS) circuitry in the past. Recently, metal-insulator-transition materials have been used to realize artificial neurons. Although memristors have been implemented to realize synaptic behavior, not much work has been reported regarding the neuronal response achieved with these devices. In this work, we use the volatile threshold switching behavior of a vertical-MoS_2_/graphene van der Waals heterojunction system to produce the integrate-and-fire response of a neuron. We use large area chemical vapor deposited (CVD) graphene and MoS_2_, enabling large scale realization of these devices. These devices can emulate the most vital properties of a neuron, including the all or nothing spiking, the threshold driven spiking of the action potential, the post-firing refractory period of a neuron and strength modulated frequency response. These results show that the developed artificial neuron can play a crucial role in neuromorphic computing.

## Introduction

Understanding the complex and overwhelming functioning of the human brain has always fascinated scientists and researchers for biological fields and beyond. The way the human brain can process huge amounts of data and handle pattern recognition with relative ease is immensely compelling and its complexity is continuously evolving. The building blocks in the brain consist of interconnected neurons and synapses, which cooperate efficiently to process incoming signals and decide on outgoing actions. Inspired by the operation of the human brain, various neuromorphic devices, circuits, and systems have been developed that can work analogous to the brain. Owing to the low power dissipation and characteristics that are similar to biological neurons, spiking neural networks (SNN) have garnered huge interests for mimicking and closely resembling the neuro-biological system^1^. SNNs, the third generation of neural network models, have been found to be more hardware friendly and energy efficient; thus making them more biologically realistic compared to other neural networks^2^. The key components of an SNN are artificial neurons and synapses where the synapses connect the pre-neurons and the post-neurons. SNN uses discrete rather than continuous spikes along with the incorporation of the concept of time. It involves efficient transfer of information based on precise timing of a sequence of spikes. Important synaptic behaviors such as synaptic plasticity, long term potentiation and depression (LTP and LTD), and Spike Time dependent Plasticity (STDP) have been studied using emerging devices such as memristors for information processing, pattern recognition and learning methods of SNN^3–5^. But, neuronal response has mostly been realized using complex CMOS circuitry, which requires several transistors to mimic a single neuron. These circuits involve a large number of active components, increasing the power dissipation and making high density integration difficult^5–7^. To overcome these issues, various emerging devices have been used to emulate a biological neuron behavior. Insulator to metal transition (IMT) in materials such as vanadium dioxide (VO_2_)^8,9^ and niobium dioxide (NbO_2_)^10^, chalcogenide-based phase change materials^11^ and magnetoelectric switching of ferromagnets such as bismuth ferrite (BiFeO_3_)^1^, have been considered to develop various types of artificial neurons. VO_2_ has a very low critical temperature of ~340 K above which its switching behavior disappears, severely limiting the operational range of devices and making them unsuitable for on-chip integration^8,9,12^. In case of phase change materials-based neurons, the degree to which the neuronal characteristics can be tuned with nominal circuitry is limited by the fact that switching properties arise from the physics of crystallization^11^. For the same reason, the switching speed in phase change neuron is slow. Threshold switching in memristors can be a natural choice for realizing artificial neurons. However, the exploitation of threshold switching in memristors to realize artificial neurons is relatively unexplored. Recently, a Ag/SiO_2_/Au threshold switching memristor (TSM) was used to demonstrate an integrate-and-fire neuron^13^. The observation of volatile threshold switching in MoS_2_ stimulates the possibility of employing this phenomenon for the realization of artificial neurons^14^. However, the switching in a lateral MoS_2_ film mediated by the grain boundaries occludes scaling of lateral device dimensions. Hence, realizing such characteristics using a vertical structure can play a critical role in improving scaling opportunities.

In this paper, we have constructed an integrate-and-fire artificial neuron by exploiting the threshold switching behavior in CVD-grown vertical MoS_2_ (v-MoS_2_) layers. A novel device structure was fabricated by growing v-MoS_2_ on a CVD-graphene monolayer as the bottom electrode and with Ni as the top contact to v-MoS_2_. The key characteristics of a biological neuron including an all or nothing spiking, a threshold driven spiking, a post firing refractory period and an input strength modulated frequency response were observed. The developed v-MoS_2_/graphene TSM artificial neuron can operate over a wide range of temperature and can potentially be scaled down to nanometer scale cross bar structures. This platform provides an attractive prospect for neuromorphic circuits, which can be seamlessly integrated with existing fabrication technologies enabling large scale realization of such devices.

**Figure 1:**
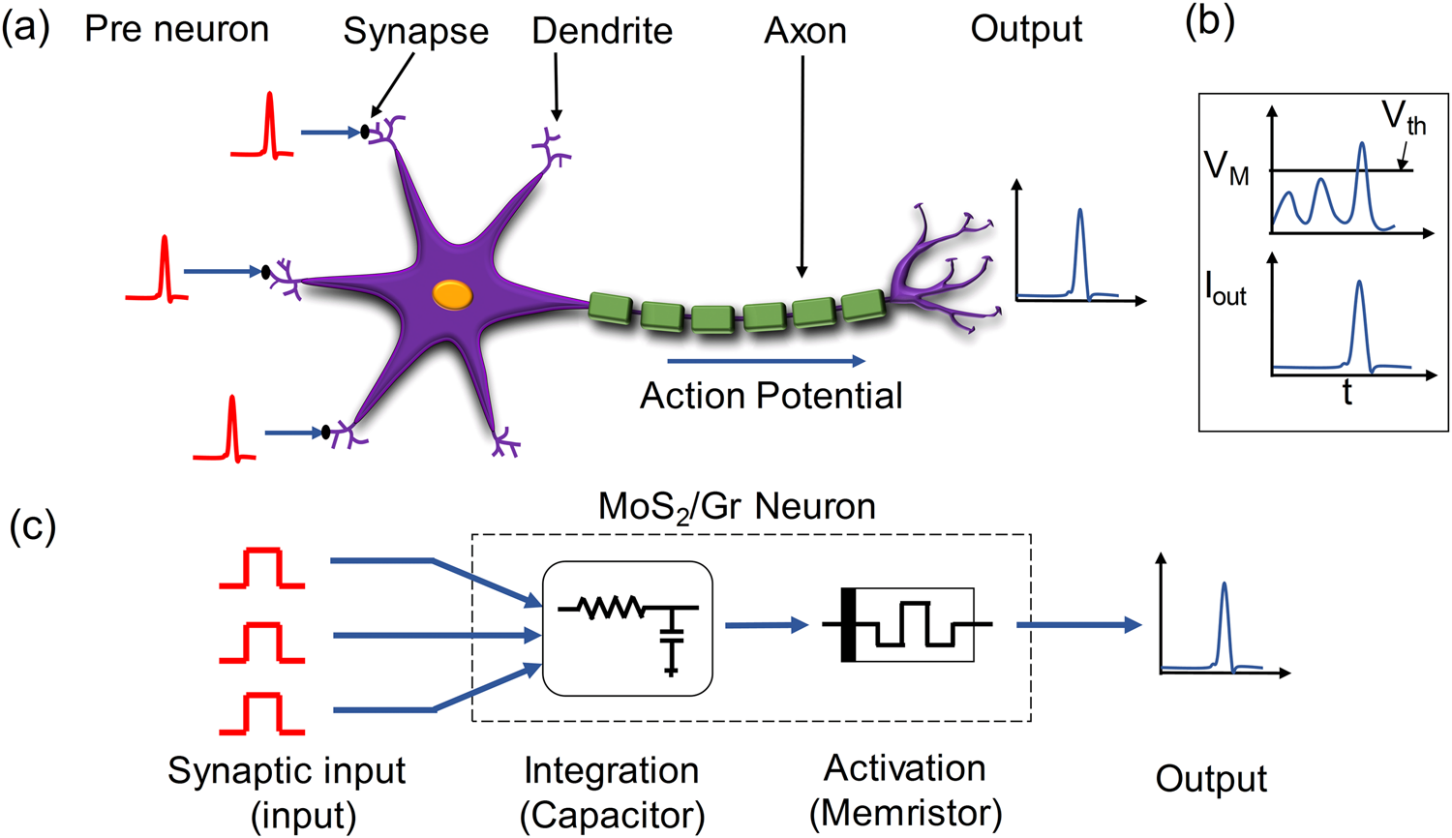
(**a**) Representation of a biological neuron from pre-neuron to output. (**b**) Output spiking of neuron with respect to the input threshold. (**c**) Conceptual representation of the v-MoS_2_/graphene memristor-based artificial neuron.

The schematic of a biological neuron connected to multiple synapses is shown in Fig. 1a. Biological neurons are connected to each other through synapses. Depending upon the input signals received by the dendrites, an action potential (also referred to as a neuron spike) is generated by the soma of the neuron, which is then passed by the axon to other neurons through synapses. The electrical equilibrium is maintained by the movement of various ions such as Na^+^, K^+^, Cl^−^ and Ca^2+^ in and out of the neuron cell. The potential difference between the interior and the exterior of the neuron is referred to as the membrane potential^1^. As the membrane potential (V_M_) builds up, the neuron produces an output spike (I_out_) when the potential reaches a particular threshold voltage (V_th_)^15,16^. This phenomenon of neuron spiking is shown in Fig. 1b. The neuron does not fire immediately after producing a spike even if it keeps receiving input signals. This period is known as the refractory period of a neuron^8,17^. After the refractory period, the neuron starts integrating the input again and prepares for the following spike.

One of the most commonly used neuron models in computational neuroscience is the integrate-and-fire (IF) model^18^. An IF artificial neuron fires as a function of the electrical potential developed across it, thus mimicking the crucial behavior of a biological neuron. Here, this behavior has been realized with a v-MoS_2_/graphene TSM as shown in the conceptual scheme in Fig. 1c. The v-MoS_2_/graphene neuron integrates the input signals received with the help of a capacitor. The capacitor integrates the charge and as soon as the voltage across the capacitor increases beyond the threshold value of the TSM, the neuron fires and an output spike is produced.

**Figure 2:**
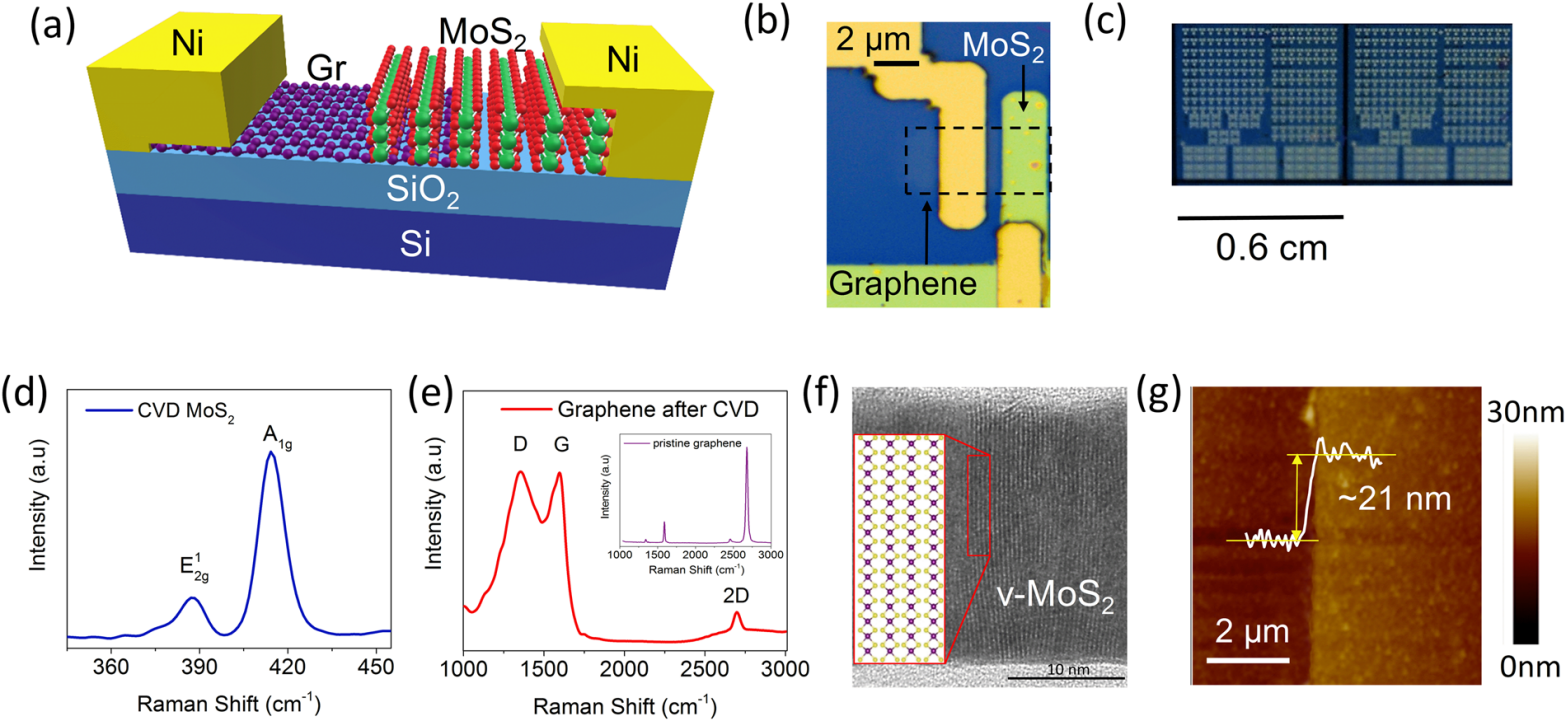
(**a**) Schematic of a v-MoS_2_/graphene TSM. (**b**) Optical image of the MoS_2_/graphene TSM. (**c**) A representative picture of a chip containing ~200 MoS_2_/graphene TSMs. (**d**) Raman spectrum of the as-grown MoS_2_ on graphene. (**e**) Raman spectrum of the graphene after CVD and of pristine graphene (inset). (**f**) HRTEM image of the cross-section of the v-MoS_2_/graphene interface showing vertical growth of MoS_2_. (**g**) AFM height image of the MoS_2_ showing the thickness of the MoS_2_ to be ~21 nm.

The device schematic of the v-MoS_2_/graphene device is shown in Fig. 2a. It consists of a CVD-grown monolayer graphene, wet transferred on the Si/SiO_2_ substrate^19^, followed by patterned growth of v-MoS_2_ on graphene. Nickel contacts were deposited on the graphene and on the v-MoS_2_. The optical image of the device is shown in Fig. 2b. A representative image of a block of a single chip with ~200 MoS_2_/graphene Threshold Switching Memristors (TSMs) is shown in Fig. 2c. The composition of the two layers was confirmed by Raman spectroscopy with excitation wavelength of 532 nm laser in ambient conditions. The spectrum in Fig. 2d was obtained on the as-grown v-MoS_2_ film over graphene in the device. Two bands corresponding to the in-plane E^1^_2g_ mode and the out-of-plane A_1g_ mode were observed at 383 cm^−1^ and 411 cm^−1^ respectively. The presence of these two distinct high intensity peaks and the difference of about 28 cm^−1^ between their two positions signifies the presence of good quality multilayer MoS_2_^20^. On pristine graphene, as shown in the inset of Fig. 2e, the Raman spectrum yielded two major distinct peaks, *viz*. the G band at 1590 cm^−1^, and the 2D band at 2690 cm^−1^. The peak intensity ratio I_2D_/I_G_ is about ~2, indicating that the monolayer graphene used for our devices is of good quality^21^. After sulfurization, the spectrum, presented in Fig. 2e, exhibits three distinct peaks: The D band at 1350 cm^−1^, the G band at 1590 cm^−1^ and the 2D band at 2690 cm^−1^. A significant decrease in intensity of the 2D band with respect to the G band is revealed. This reduced value of the peak intensity ratio of the G and 2D peak and the large D band indicate the introduction of defects in the graphene layer during sulfurization^22^. The detailed analysis of the v-MoS_2_/graphene 2D-2D van der Waal’s heterojunction was carried out by High Resolution Transmission Electron Microscopy (HRTEM). Figure 2f shows the cross-section HRTEM image of 2D MoS_2_ on graphene/Si/SiO_2_. It can be clearly observed that MoS_2_ grows vertically on the graphene surface with high density of exposed edge planes. The vertical orientation of 2D MoS_2_ atomic layers was obtained by the sulfurization of thick Mo films, which essentially minimizes the strain energy arising as a result of volume expansion during Mo to MoS_2_ conversion^23^. This eventually leads to the formation of polycrystalline MoS_2_ structure with vertically orientated grains perpendicular to the substrate. The thickness of the CVD grown v-MoS_2_, as determined by using Atomic force microscopy (AFM) (Fig. 2g), was 21 nm, which is in agreement with the thickness observed in the cross-section TEM analysis of Fig. 2f.

## Results and Discussion

### DC Characteristics

**Figure 3:**
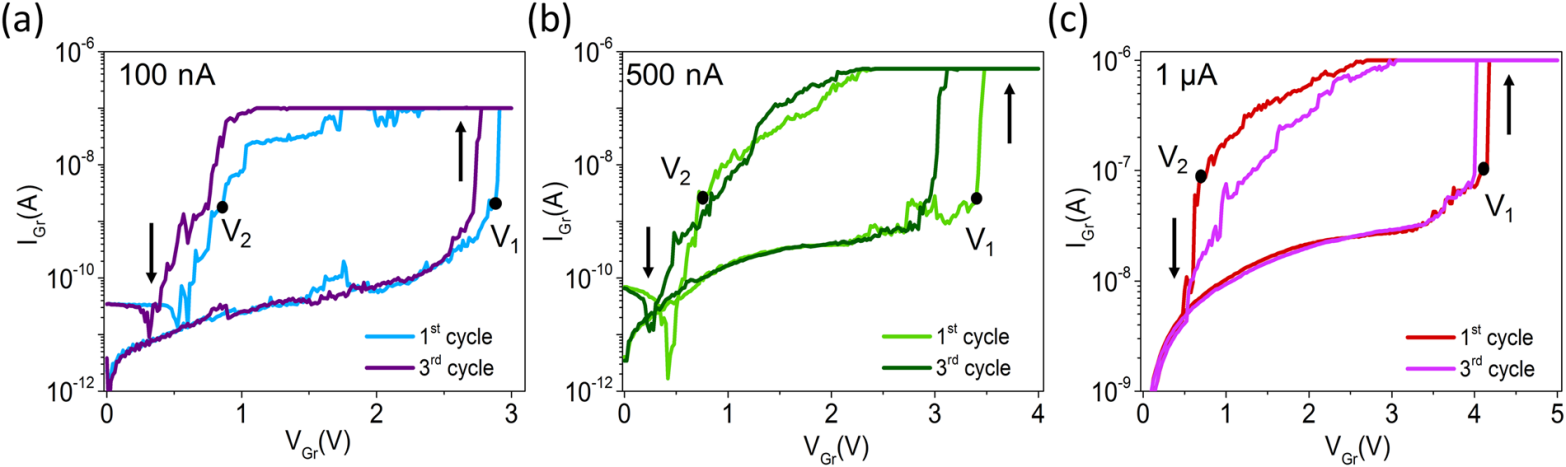
I-V characteristics of a single v-MoS_2_/graphene device showing volatile behavior over multiple cycles with current compliance of (**a**) 100 nA. (**b**) 500 nA. (**c**) 1 µA when the voltage on the graphene (Gr) electrode is swept keeping the other electrode at 0 V in all cases.

To assess the switching characteristics with respect to the current compliance, the v-MoS_2_/graphene TSM was tested at various current compliances of 100 nA, 500 nA, and 1 µA as shown in Fig. 3a–c. The I-V characteristics of the same device were recorded by sweeping the voltage on the graphene electrode while keeping the MoS_2_ electrode at 0 V first with a current compliance of 100 nA repeatedly over 3 cycles. This process is then repeated with a current compliance of 500 nA and 1 µA. In all the cases, it is observed that the device is initially in its high resistance state (HRS) until a voltage higher than the threshold voltage is applied. At the onset of the threshold voltage (V_1_), the device undergoes an abrupt transition from the HRS to a low resistance state (LRS). During the reverse sweep, the device reverts to HRS as the voltage reduces below a particular value (V_2_). It is interesting to note that the device retains the volatile threshold switching behavior for all the applied current compliances.

### Spiking characteristics

**Figure 4:**
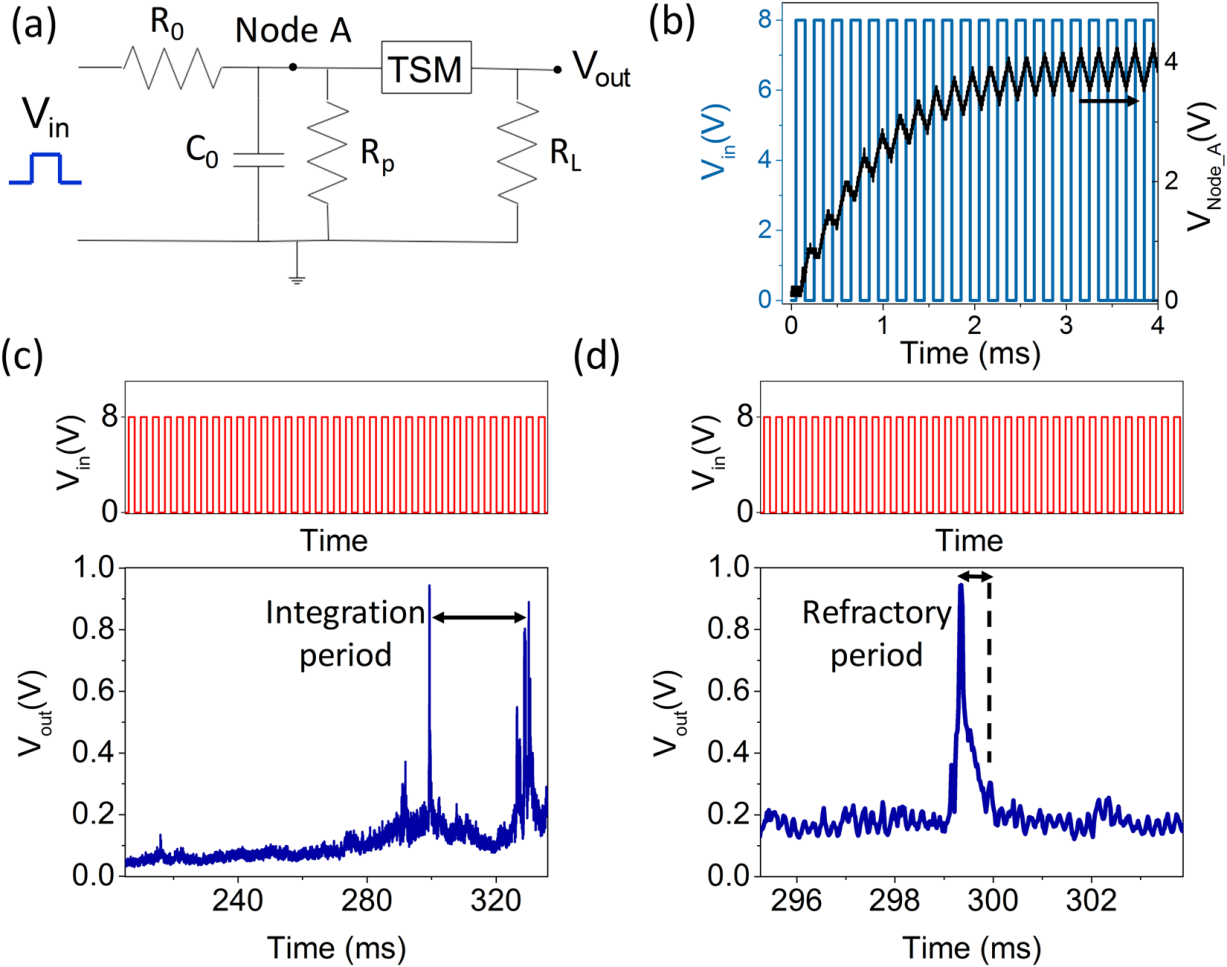
(**a**) Schematic of the circuit used to realize the artificial neuron. (**b**) Input voltage pulses and voltage at node A as a function of time. When a series of voltage pulses of an amplitude of 8 V with T_ON_ = 100 µs (blue line) are applied as input to the circuit, the capacitor C_o_ starts charging and the voltage at node A (black line) increases till the capacitor gets completely charged. (**c**) (Top) Input voltage pulses of amplitude 8 V, T_ON_ = 100 µs, frequency = 5 kHz (not to scale). (Bottom) Output spike of the artificial neuron showing the integration time. (**d**) (Top) Input voltage pulses of amplitude 8 V, T_ON_ = 100 µs, frequency = 5 kHz (not to scale). (Bottom) Output spike of the artificial neuron showing the refractory period of the neuron.

After confirming the volatile threshold switching behavior of v-MoS_2_/graphene device, we now concentrate on the demonstration of an artificial neuron exploiting the volatile switching. The schematic of the circuit used for realizing the artificial neuron with the v-MoS_2_/graphene TSM is shown in Fig. 4a. The TSM device is connected in parallel with a resistor, R_p_ = 1 MΩ and a capacitor C_o_ = 100 nF, which are connected in series to a resistor R_o_ = 10 kΩ. A load resistor R_L_ = 1 MΩ is connected in series with the TSM to measure the output voltage. The resistors and the capacitor are connected to the device externally through a breadboard. The parallel resistor ‘R_p_’ is connected to drain excessive current away from the device, thereby protecting the device. Its value is chosen to be 1 MΩ which is less than the R_LRS_ = ~3 MΩ and greater than R_o_ such that C_o_ does not discharge through R_p_. The input source is a train of constant amplitude voltage pulses applied to the left node of the circuit with frequency 5 kHz and T_ON_ = 100 µs. The output voltage is measured across the R_L_. In Fig. 4b, when a series of input pulses (blue line) of an amplitude of 8 V with T_ON_ = 100 µs are applied to the circuit, the capacitor starts charging and the voltage starts increasing at node A (black line) till the capacitor gets completely charged. Initially, the TSM is in its HRS and the device resistance ‘R_HRS_’ is high (~10 MΩ) compared to R_o._ The charging time constant (R_o_C) is ~100 times lower than that for the discharging time constant [(R_P_||R_HRS_|| R_L_) C_o_], hence the leakage through the device is almost negligible. Therefore, on application of the input pulses to the circuit, the capacitor (C_o_) gets charged. As the charge across the capacitor increases and the voltage at node A reaches the threshold voltage of the TSM, the device switches from the HRS to the LRS. Since the TSM is now in the LRS state, the capacitor starts discharging through the device and an abrupt increase in the current appears at the rightmost node (output). This corresponds to the increase in voltage at t ≈ 300 ms, observed in Fig. 4c. Now, the capacitor starts discharging and consequently the voltage at node A starts dropping down. Once the voltage at node A decreases, the TSM device reverts to the HRS. This results in the output current and voltage to reduce as well, causing a spike in the output voltage (Fig. 4c). The capacitor starts charging again signifying commencement of the integration period of the neuron as shown in Fig. 4c. The process gets repeated to produce the subsequent spikes shown in the figure at t > 320 ms. The charging and discharging of the capacitor, manifested through the voltage at node A, are shown in Figs S1 and S2 (Supplementary Information) for different spiking events.

During the timeframe when the neuron is firing, the net charge integration across the capacitor is essentially zero. This is because any input voltage pulse applied is being directly drained by the TSM device (it being turned on). This period emulates the post firing refractory period of a biological neuron^1,8,13^. Once the refractory period is over, the device reverts from the LRS state to its HRS and the capacitor ‘C_o_’ starts integrating again. The refractory period from the v-MoS_2_/graphene TSM is shown in Fig. 4d and it is further corroborated in terms of the voltage at Node A as shown in Fig. S3.

**Figure 5:**
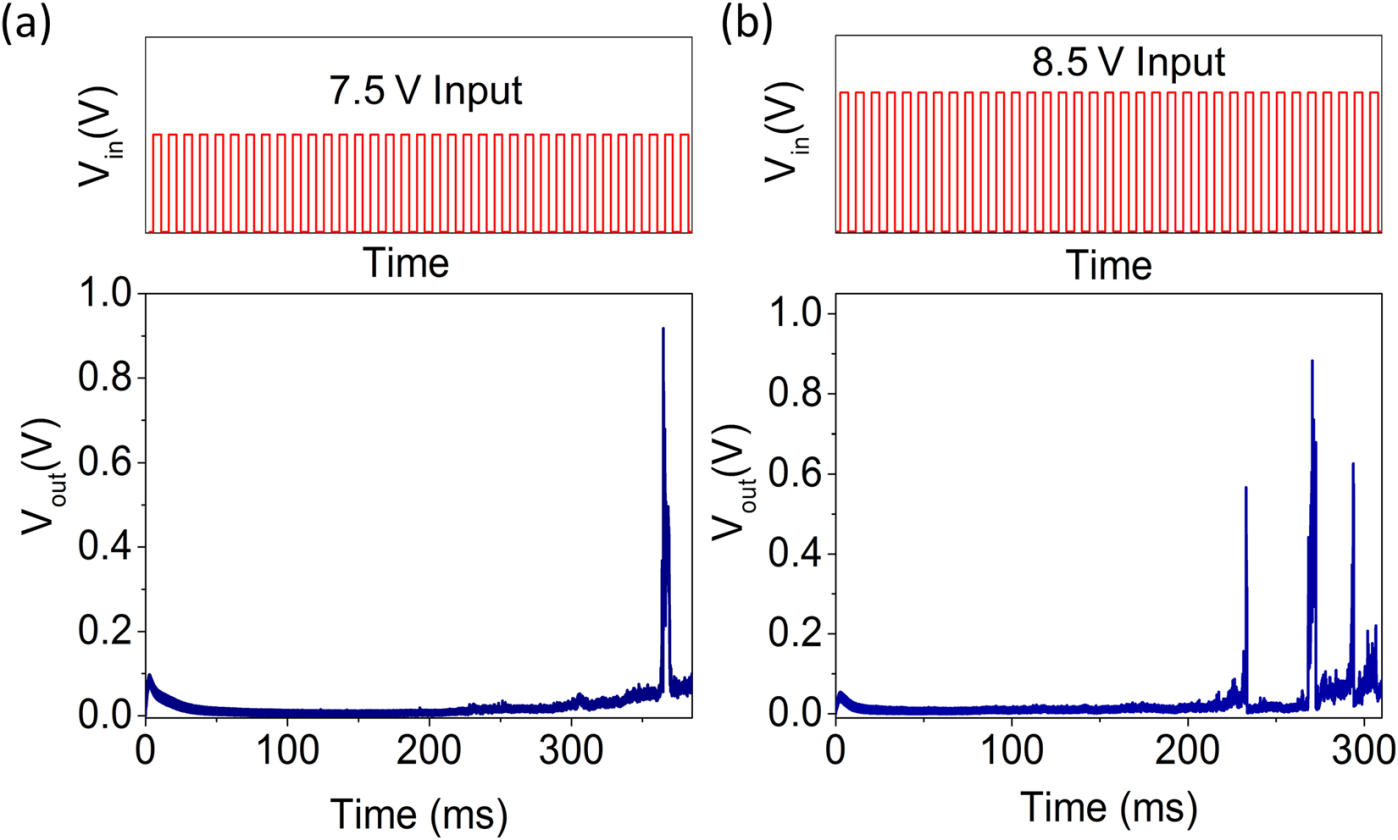
Strength-modulated frequency response for v-MoS_2_/graphene TSM. (**a**) (Top) Input voltage pulses of amplitude 7.5 V, T_ON_ = 100 µs, frequency = 5 kHz (not to scale). (Bottom) Single output spike observed for input voltage pulses of amplitude 7.5 V. (**b**) (Top) Input voltage pulses of amplitude 8.5 V, T_ON_ = 100 µs, frequency = 5 kHz (not to scale). (Bottom) Three output spikes observed for input voltage pulses of amplitude 8.5 V.

Another key characteristic of a biological neuron is that the frequency of spiking increases with higher strength of the input^13^. To emulate this behavior using the developed artificial neurons, a train of pulses with the same frequency 5 kHz (T_on_ = 100 µs) but two different amplitudes are applied to the v-MoS_2_/graphene neurons. On application of input pulses of 7.5 V to the circuit (Fig. 5a), a potential drop across R_o_ occurs, leading to a voltage of ~4 V at the node A and a single output voltage spike is observed. On the other hand, increasing the amplitude of the input pulses to 8.5 V (Fig. 5b), three spikes are recorded in a shorter time range. The spiking frequency increases for input with a higher amplitude as seen in Fig. 5. In case of input pulses with a lower voltage, the capacitor requires a higher number of pulses to trigger the output spike than in case of input pulses with a higher voltage. Hence, the input strength modulated frequency dependence characteristics can be emulated by the developed v-MoS_2_/graphene artificial neurons.

### Stochastic switching behavior

**Figure 6:**
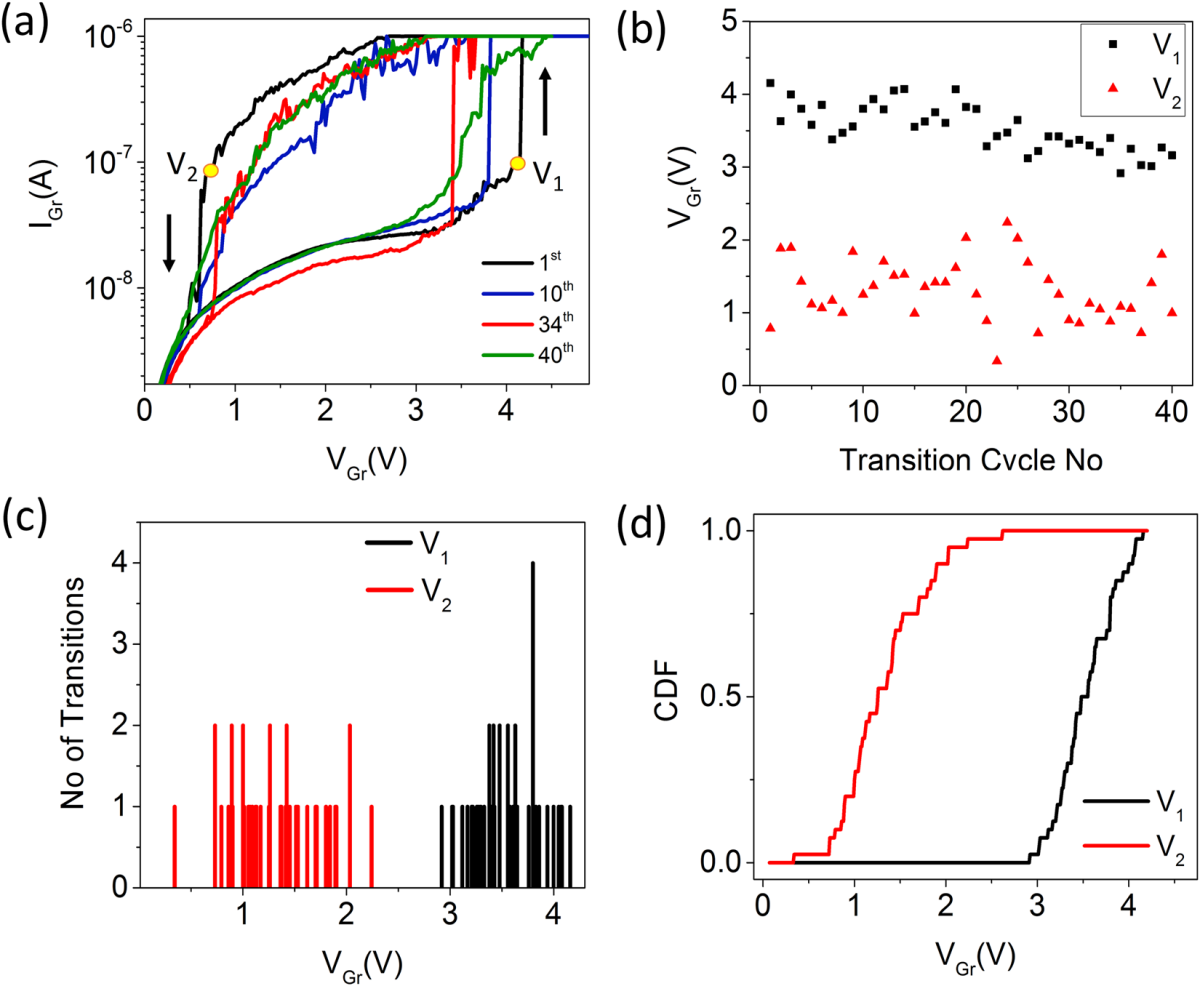
(**a**) I-V characteristics of v-MoS_2_/graphene device over 40 cycles. (**b**) Variation of V_1_ and V_2_ over 40 cycles. (**c**) Number of HRS-LRS and LRS-HRS transitions corresponding to a particular V_1_ and V_2_, respectively. (**d**) Cumulative distribution function of V_1_ and V_2_.

We studied the DC characteristics of the TSM for over 40 cycles to shed light on the nature of the switching voltages. It is observed that the device retained the abrupt transition from the HRS to the LRS over this period as shown in Fig. 6a. The threshold voltage at which the device switches from the HRS to the LRS (V_1_) and from the LRS to the HRS (V_2_) are plotted over the 40 cycles shown in Fig. 6b. It is evident from Fig. 6c that the window between V_1_ and V_2_ is maintained over the cycling period. Also, it is observed that V_1_ and V_2_ are probabilistic in nature and follow a stochastic distribution. Figure 6d shows the cumulative distribution function for the switching probability of the v-MoS_2_/graphene TSM, which, in turn, produces the neuron spiking probability. Additionally, the stochastic switching behavior of the v-MoS_2_/graphene TSM originating from device level stochasticity makes these probabilistic spiking neurons viable for applications in the field of hardware security, such as Random Number Generators (RNGs)^24,25^.

### Switching mechanism

Recent reports have shown that the switching mechanism in single-layer MoS_2_ can be attributed to the grain boundaries (GBs)^14^. The switching behavior of our v-MoS_2_/graphene devices can be attributed to the multiple grain boundaries in the polycrystalline films of v-MoS_2_. To further comprehend the switching mechanism in the v-MoS_2_/graphene devices, the DC measurements were carried out in vacuum. It is interesting to note that in the absence of oxygen, the fabricated devices do not show the volatile switching behavior (Fig. S4, supporting information) that was observed in the presence of oxygen. It can be further noted that the switching window disappears and the device does not reset to its HRS during the reverse voltage sweep. Hence, the migration of oxygen ions facilitated by the grain boundaries in the v-MoS_2_ is the probable reason the switching mechanism in the realized v-MoS_2_/graphene devices. The vacuum ambient removes the oxygen ions from the system completely.

## Conclusion

In summary, we have demonstrated a novel 2D material-based TSM comprising v-MoS_2_ layers grown on a monolayer graphene template. The device mimics the characteristics of a biological neuron via volatile resistive threshold switching behavior. The developed IF artificial neuron exhibits the key behaviors of a biological neuron which include an all or nothing spiking, a threshold driven spiking of the action potential, a post-firing refractory period of a neuron and a strength modulated frequency response. These properties coupled with the stochastic threshold switching behavior mediated by oxygen ion migration along the vertical grains of MoS_2_ make these artificial neurons viable for applications in real time computing systems based on event spiking such as pattern recognition, neuromorphic vision sensors, and hardware security. While the current through the v-MoS_2_/graphene TSM is presently low (~1 μA) demanding high input voltages for the circuit operation, the v-MoS_2_ stack can be engineered in the future by modifying its thickness and growth conditions to allow larger currents to flow through the circuit. This will consequently reduce the voltage requirements of the circuit as well.

## Methods

### Device fabrication

First, commercially purchased CVD-grown monolayer graphene was wet transferred over a Si/SiO_2_ (300 nm SiO_2_) substrate. The graphene was then patterned by photolithography and etched by oxygen plasma. The sample was again patterned and 10 nm thick Mo films were deposited on the etched graphene using an electron beam (e-beam) evaporation system (Temescal FC-2000). The Mo films were subsequently sulfurized to MoS_2_ in a low-pressure CVD furnace. 50 nm Ni contacts on both graphene and MoS_2_ were patterned, e-beam evaporated and lifted off.

### CVD Growth

The patterned Mo film on graphene/Si/SiO_2_ substrate were loaded in the center of a quartz tube CVD furnace with a ceramic boat having sulfur powder at the upstream of Mo sample. The furnace was pumped down to a base pressure of ≤1 mTorr and then purged with argon (Ar) gas. The furnace was heated up to a temperature of ∼650−700 °C with a constant flow of 100 SCCM (standard cubic centimeters per minute) Ar gas at a pressure of ∼100 mTorr. After a 30-min reaction time, the furnace was naturally cooled down and the substrate was taken out of the furnace. The change of color of the substrate from blue to dark green, indicates the sulfurization of Mo on the graphene.

### Raman, AFM and TEM characterization

The Raman spectra were obtained using a confocal Raman system (WITEC Alpha 300RA) in ambient conditions with a 532 nm laser source for excitation. The laser was focused with a Zeiss 50× objective lens (numerical aperture of 0.7) with power ~5.7 mW and integration time 5 s. Each raman spectra were taken over 10 scans. The AFM topography was acquired on an Anasys NanoIR2 system in tapping mode using Anasys tapping mode AFM probes (Model No. PR-EX-T125-10). The TEM imaging was carried out using JEOL ARM-200F equipped with an aberration corrector which provides spatial resolution down to ~1 Å at an operating voltage of 200 kV.

### Electrical characterization

The electrical measurements were carried at room temperature in Micromanipulator 6200 probe station using a Keysight B1500A semiconductor device analyzer along with WGFMUs for pulse I-V measurements. The vacuum measurements were carried out in a Janis cryo-probe station. A Tektronix DPO 2024B oscilloscope was used to measure the node voltage.

## Electronic supplementary material

Supplementary Information

## Data Availability

All data generated and analyzed during this study are either included in the published article itself (or available within the Supplementary Information files).
