# Supplementary material for: Artificial Neuron using Vertical MoS2/Graphene Threshold Switching Memristors
Source: Sci Rep. 2019 Jan 10;9:53. doi: 10.1038/s41598-018-35828-z (PMC6328611; doi:10.1038/s41598-018-35828-z)
Supplement: Supplementary file 1 — Supplementary Information [file 41598_2018_35828_MOESM1_ESM.docx]

**Supplementary Information**

Artificial Neuron using Vertical MoS_2_/Graphene Threshold Switching Memristors

*Hirokjyoti Kalita,^1,2^* ^†^ *Adithi Krishnaprasad,^1,2^*^†^ *Nitin Choudhary,^1^*^†^ *Sonali Das,^1^ Durjoy Dev,^1,2^Yi Ding^1,3^, Laurene Tetard,^1,4^ Hee-Suk Chung,^5^ Yeonwoong Jung^1,2,3^, and Tania Roy^1,2,3*^*

*^1^NanoScience Technology Center, University of Central Florida, Orlando, Florida 32826, USA.*

*^2^Department of Electrical and Computer Engineering, University of Central Florida, Orlando, Florida 32816, USA*

*^3^Department of Materials Science and Engineering, University of Central Florida, Orlando, Florida 32816, USA.*

*^4^Department of Physics, University of Central Florida, Orlando, Florida 32816, USA.*

*^5^Analytical Research Division, Korea Basic Science Institute, Jeonju, Jeollabuk-do, 54907, South Korea*

^†^*Authors have equal contribution.*

^*^*Corresponding author:* [*tania.roy@ucf.edu*](mailto:tania.roy@ucf.edu)

**S1. v-MoS_2_/graphene artificial neuron spike-firing process**

To understand the process by which the v-MoS_2_/graphene TSM fires upon application of input voltage pulses, we recorded the voltage across capacitor by measuring the voltage at node A of Figure 4a in the main text. Initially when the device is in the HRS at t = 0, the capacitor is charging and the charging time constant is given as T_C_ = R_o_ * C_o_ where R_o_ = 10 kΩ. In case of discharging, the discharging time constant is given as T_D_ = ((R_P_ **||** R_LRS_ **||** R_L_) * C_o_). In the discharging loop, the equivalent resistance is very high on account of R_P_ = R_L_ = 1 MΩ and R_LRS_ = ~3 MΩ, hence T_D_ >> T_C_.

Figure S1 shows an output voltage spike of a typical v-MoS_2_/graphene artificial neuron and the corresponding voltage change at node A as a function of time. As the input pulses are applied, the capacitor C_o_ charges and node A steadily reaches a voltage higher than the threshold voltage of the v-MoS_2_/graphene TSM (V_1_ - indicated in Figure 3 of the main text). At t = 1.5 s, the v-MoS_2_/graphene TSM switches from the HRS to the LRS, resulting in an increase in output voltage and current. Now, the capacitor starts discharging through the TSM but it is not able to discharge completely since T_D_ >> T_C_. The voltage at node A drops by ~100 mV during the discharging process of the capacitor. The capacitor starts charging again without being able to discharge completely due to the competing charging and discharging processes. The output voltage of the neuron drops during the discharging process. This results in the spike in V_out_ of Figure S1 (top).

**

**

**Figure S1. (a)** The output spike obtained from the v-MoS_2_/graphene artificial neuron. (b) The voltage variation across the capacitor (Node A) corresponding to the spike.

Figure S2 shows similar spiking in the output voltage and corresponding change in voltage at node A for another v-MoS_2_/graphene device. At t = 0.44 s, the TSM switches from HRS to LRS, resulting in an increased output current and voltage as seen in Figure S2a. The capacitor C_o_ starts discharging at that instant, immediately reducing the output voltage. The voltage at node A drops during the discharging process. Again, since the charging time is much lower compared to the discharging time of the circuit, the capacitor is not able to discharge completely. The voltage at node A drops by only ~70 mV. The spike in the output voltage corresponds to this dip in the voltage at node A. For successive incoming voltage pulses, the capacitor starts building charge again (corresponding to integration period of neuron), seen in the increase of voltage at node A (Figure S2b). When the voltage at node A is able to switch the TSM from HRS to LRS again, the capacitor C_o_ starts discharging again. This is seen in the increase in V_out_ and the decrease in voltage at node A at t ≈ 0.55 s. The incoming pulse is withdrawn at t = 0.57 s.

**

**

**Figure S2. (a)** The output spike obtained from a v-MoS_2_ artificial neuron, different from that shown in Fig. S1. (b) The voltage variation across the capacitor (as measured from the voltage at Node A) corresponding to the neuron’s spike.

The low drop of voltage at node A corresponding to the spike of the neuron is a result of the high resistance of the TSM device in its LRS. Since the TSM allows only ~1 μA of current to flow through it, the resistance of the device is in MΩ, increasing the discharging time of the circuit. To obtain a large voltage drop at node A as a consequence of the complete discharge of the capacitor Co, T_D_ must be much lower than T_C_^1^.

**S2. Refractory Period of v-MoS_2_/Graphene artificial neuron**

The refractory period is the period when the neuron doesn’t fire immediately after firing even if it receives an input signal until the voltage is completely drained. During this period when the neuron is firing even if the artificial neuron is receiving input pulses (T_on_=100 µS), the voltage is drained first as the MoS_2_/graphene TSM is in its LRS and then the capacitor starts integrating. This can be seen in Figure S3a which shows refractory of period of a neuron spiking and Figure S3b which shows the corresponding voltage change at node A. From Figure S3a, it is clear that after the spiking, the v-MoS_2_/graphene artificial neuron the first drains the voltage and the capacitor starts integrating the charge as seen from the increasing voltage at Node A as indicated in Figure S3b once the refractory period is complete.





**Figure S3. (a)** The output spike obtained from a v-MoS_2_/Graphene artificial neuron, showing the refractory period. (b) The voltage across the capacitor (as measured from Node A) corresponding to the refractory period of the v-MoS_2_/Graphene artificial neuron.

**S3. DC Characteristics of v-MoS_2_/Graphene threshold switching memristors in vacuum**

The I-V characteristics of a v-MoS_2_/graphene threshold switching memristor (TSM) is measured in vacuum at a pressure of ~8x10^-4^ mbar with a current compliance of 1 µA. For the first cycle, the device shows volatile threshold switching characteristics. But as the voltage is swept for consecutive cycles, it is observed that the device loses its volatile switching behavior, as shown in Figure S4. The v-MoS_2_/graphene TSM does not return to its high resistance state (HRS) as the voltage is swept back and remains in its low resistance state (LRS), which is indicative of the fact that oxygen plays a crucial role in the volatile switching of these devices.

**

**

**Figure S4.** The I-V characteristics of a representative v-MoS_2_/Graphene device measured in vacuum. As the voltage is swept multiple times, the device does not exhibit the volatile switching behavior in the absence of oxygen.

**S3. DC Characteristics of v-MoS_2_/Graphene threshold switching memristors in vacuum**

The output current was measured with the help of the Semiconductor Parameter Analyzer (Keysight B1500A) along with WGFMUs (Waveform Generation and Fast Measurement Units). The WGFMUs are used to generate the input pulses and measure the fast output current response. The resistor and capacitor were connected to the device using a breadboard while a probe station was used to probe the v-MoS_2_/Graphene TSM. A representative image of the experimental setup is shown in Figure S5.


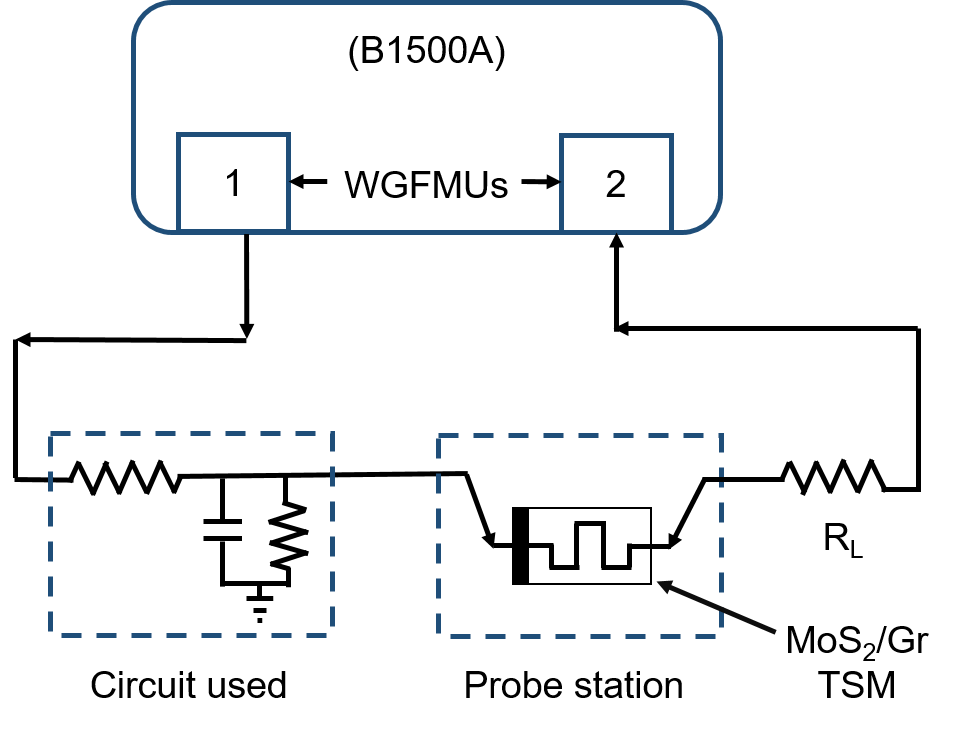


**Figure S5.** The Experimental setup used to obtain the response of the artificial neuron.

REFERENCES

1 Zhang, X. *et al.* An Artificial Neuron Based on a Threshold Switching Memristor. *IEEE Electron Device Lett.* **39**, 308-311 (2018).
